# Overcoming floor and ceiling effects in knee arthroplasty outcome measurement: mapping the Oxford Knee Score and High Activity Arthroplasty Score onto a common scale

**DOI:** 10.1302/2046-3758.1210.BJR-2022-0457.R1

**Published:** 2023-10-04

**Authors:** Conrad J. Harrison, Constantin Y. Plessen, Gregor Liegl, Jeremy N. Rodrigues, Shiraz A. Sabah, David J. Beard, Felix Fischer

**Affiliations:** 1 Surgical Intervention Trials Unit, Nuffield Department of Orthopaedics, Rheumatology and Musculoskeletal Sciences, University of Oxford, Oxford, UK; 2 Department of Psychosomatic Medicine, Center for Internal Medicine and Dermatology, Charité – Universitätsmedizin Berlin, corporate member of Freie Universität Berlin, Humboldt-Universität zu Berlin, and Berlin Institute of Health, Berlin, Germany; 3 Clinical Trials Unit, University of Warwick, Coventry, UK; 4 Department of Plastic Surgery, Stoke Mandeville Hospital, Buckinghamshire Hospitals NHS Trust, Aylesbury, UK

**Keywords:** Oxford Knee Score, High Activity Arthroplasty Score, Common metric, Common scale, Item response theory, knee arthroplasty, knee, arthroplasty, patient-reported outcome measures (PROMs), partial knee arthroplasty, Patient-Reported Outcomes Measurement Information System, root means square, clinicians, covariance

## Abstract

**Aims:**

To map the Oxford Knee Score (OKS) and High Activity Arthroplasty Score (HAAS) items to a common scale, and to investigate the psychometric properties of this new scale for the measurement of knee health.

**Methods:**

Patient-reported outcome measure (PROM) data measuring knee health were obtained from the NHS PROMs dataset and Total or Partial Knee Arthroplasty Trial (TOPKAT). Assumptions for common scale modelling were tested. A graded response model (fitted to OKS item responses in the NHS PROMs dataset) was used as an anchor to calibrate paired HAAS items from the TOPKAT dataset. Information curves for the combined OKS-HAAS model were plotted. Bland-Altman analysis was used to compare common scale scores derived from OKS and HAAS items. A conversion table was developed to map between HAAS, OKS, and the common scale.

**Results:**

We included 3,329 response sets from 528 patients undergoing knee arthroplasty. These generally met the assumptions of unidimensionality, monotonicity, local independence, and measurement invariance. The HAAS items provided more information than OKS items at high levels of knee health. Combining both instruments resulted in higher test-level information than either instrument alone. The mean error between common scale scores derived from the OKS and HAAS was 0.29 logits.

**Conclusion:**

The common scale allowed more precise measurement of knee health than use of either the OKS or HAAS individually. These techniques for mapping PROM instruments may be useful for the standardization of outcome reporting, and pooling results across studies that use either PROM in individual-patient meta-analysis.

Cite this article: *Bone Joint Res* 2023;12(10):624–635.

## Article focus

- Researchers and clinicians can choose from many different patient-reported outcome measures (PROMs) to assess knee arthroplasty outcomes.
- Using item response theory, it is sometimes possible to map scores from different PROMs to a common scale in order to standardize outcome measurement, meta-analyze results, and harness the relative advantages of different instruments.
- We aimed to do this with the Oxford Knee Score (OKS) and High Activity Arthroplasty Score (HAAS).

## Key messages

- The HAAS is targeted towards higher levels of knee health than the OKS.
- By combining these instruments, we can achieve a more precise measurement than is possible from either instrument alone, particularly at higher levels of knee health.
- Using our conversion table, readers can map scores from either instrument to a common scale.

## Strengths and limitations

- We used robust item response theory parameters for the OKS, generated previously from registry data involving over 350,000 participants, but the HAAS parameters presented here were based on a smaller sample of 528 participants, each with up to seven repeated measures that we treated independently.
- Our conversion table has not been externally validated using individual patient data, and this should be a focus for future research.

## Introduction

Patient-reported outcome measures (PROMs) are increasingly used to measure health outcomes from the patient’s perspective.^1-3^ A recent review identified 34 different joint-specific PROMs in use following total knee arthroplasty. Nine of these PROMs had good measurement properties and quality of supporting evidence to be recommended for use.^4^ Nearly all instruments measured similar health constructs – pain, function, and/or activity. A broad selection of valid PROMs allows triallists to select the best instrument to measure the specific health construct of interest. However, the use of many different PROMs also limits the ability to meaningfully pool outcome measurements across studies and to interpret study results. For some clinical applications, an overall assessment of knee health may be more desirable.

Item response theory (IRT) is a psychometric framework that can be used to map the scores from different PROMs onto a common scale, allowing the scores from one PROM to be ‘translated’ into the scores of another, provided item response data from each PROM combine to meet certain statistical prerequisites.^5^ This technique has previously been applied to standardize the measurement of depression severity,^6^ and it has also been applied in knee health, to map scores from the Patient-Reported Outcomes Measurement Information System (PROMIS) and the Knee injury and Osteoarthritis Outcome Score (KOOS) physical function short-forms.^7^

In IRT, statistical models are used to describe the relationship between the level of health construct (e.g. knee health) and the probability of responding to an item in a given way.^8^ Each item functions independently from the others, and so when prerequisites are met, items can be combined across different questionnaires. With IRT, it is sometimes possible to combine questionnaires with complementary measurement properties onto a common scale, and this is highly relevant to the Oxford Knee Score (OKS)^9,10^ and High Activity Arthroplasty Score (HAAS).^11^

The OKS contains 12 items, each with five response options. It is typically scored by summing the responses of each item, resulting in a sum-score that ranges from 0 to 48, with a higher score indicating a better clinical state. It can also be scored using IRT, through published model parameters^12^ or an online calculator.^13^ While IRT scores are continuous, with no lower or upper bound, the knee health range measured by OKS responses extends from approximately -3.40 to 3.94 logits. These represent Z-scores which can be interpreted directly or transformed in the same manner as PROMIS T-scores.^14^ Measurements derived from IRT scoring and classical sum-scoring of the OKS share a Pearson correlation coefficient of 0.988.^12^ We have previously estimated the minimal important difference of the common (untransformed) scale as 0.584, based on the mean difference in OKS score between those who felt ‘a little better’ and ‘about the same’ following primary arthroplasty in the NHS PROMs programme.^15^ This value may vary depending on the context and method used to estimate it.

A potential limitation of the OKS is that it demonstrates a ceiling effect in patients who have undergone elective, primary knee arthroplasty, with 3.7% of patients achieving its maximum score at six months.^12^ There has been debate about this previously, and the developers of the instrument have suggested that the ceiling effect is negligible.^16^ However, our recent IRT analysis has shown that while the OKS generally provides precise and discriminatory measurement in preoperative populations, postoperative patients with higher scores (for example, sum-scores exceeding 40 or IRT Z-scores exceeding 2.5) are measured with considerably less precision.^12^ In real-world terms, the OKS would struggle to differentiate between a patient who goes back to doing their shopping and gardening after a knee arthroplasty, and a patient who is able to go back to playing competitive tennis.

The HAAS, on the other hand, is targeted towards patients with a higher level of knee health. It was developed in 2010 specifically to provide discriminatory measurement among highly functioning arthroplasty recipients. Items were developed following a review of existing instruments and consultation with patients and clinicians. In its validation study, the HAAS demonstrated internal consistency (Cronbach’s alpha 0.86) and convergent validity against the OKS, the Harris Hip Score, and the Western Ontario and McMaster Universities Arthritis Index (WOMAC), but with no ceiling effect.^11^ The HAAS contains four items, including those relating to running and climbing stairs two at a time, with four, five, six, and seven response options. Sum-scores range from 0 to 18 with a higher score again indicating a better clinical state. By combining the OKS and HAAS onto a common scale, it may be possible to mitigate the impact of the OKS ceiling effect on high-level knee health measurement following arthroplasty. A combined OKS and HAAS scale would potentially allow precise and discriminatory measurement in both preoperative and postoperative arthroplasty populations.

The aim of this study was to calibrate HAAS items onto the same IRT scale as the OKS. Doing this could support provision of conversion tables that can translate the scores of either instrument onto a common scale, aid in the pooling of study results when either PROM has been used, and allow future researchers to generate IRT scores for the combined instrument, capturing a broader spread of post-arthroplasty knee health measurements.

## Methods

### Item response theory parameters for the Oxford Knee Score

As a starting point for this work, we used previously published IRT parameters for the OKS.^12^ These were based on the preoperative responses of over 350,000 patients undergoing elective primary knee arthroplasty in NHS England between 1 April 2012 and 31 March 2020. These parameters are used in the statistical models described by IRT to derive continuous measurements from the responses to items in the OKS. They can either provide highly granular measurements that account for different patterns in responses to the items, or they can be used to approximately convert OKS sum-scores to continuous measurements known as expected a posteriori (EAP) sum-scores.^17^

### Paired Oxford Knee Score and High Activity Arthroplasty responses

We performed a secondary analysis on data from the TOPKAT study, which was a pragmatic randomized controlled trial that compared total and partial knee arthroplasty for medial compartment osteoarthritis. The trial recruited 528 patients across 27 centres in the UK, and collected responses to the OKS and HAAS at baseline, two months, and one, two, three, four, and five years post-randomization.^1^

We summarized demographics and missing data patterns through descriptive statistics, before performing IRT assumption testing with the PROM item responses. We chose to treat repeated PROM measurements independently. While this approach did not account for the potential lack of conditional independence between within-person repeated measures, it did ensure a broad range of item responses in our sample and leveraged all available data. We considered this approach preferable to using baseline-only data in this study.

### Assumption testing

We undertook assumption testing for IRT analysis following established procedures.^6^ The first step was to check whether the health constructs that might be measured by OKS and HAAS (for example pain, function, and activity) are sufficiently closely related to be considered as a single entity (knee health). To do this, we calculated the disattenuated Pearson correlation coefficient between OKS and HAAS sum-scores (the raw correlation coefficient divided by the square root of the product of Cronbach’s alpha for each scale, which mitigates the impact of measurement error on correlation),^18^ and performed a confirmatory factor analysis (CFA). We did this with the *lavaan R* package (version 0.6 to 11),^19^ using polychoric correlations and the diagonally weighted least squares estimator. We judged the following fit statistic thresholds to suggest unidimensionality: root mean squared error of approximation (RMSEA) < 0.060, standardized root means square residual (SRMR) ≤ 0.080, comparative fit index (CFI) ≥ 0.950, and Tucker-Lewis index (TLI) ≥ 0.950.^20^

We performed a Mokken analysis to test whether the scores on each item were monotonically related to the total score of all items combined. We considered Loevinger’s H_i_ values > 0.3 to demonstrate monotonicity for each item.^21^ We checked for local independence of item responses using Yen’s Q3 statistic, a measure of residual covariance. We considered a value of > 0.2 to suggest that the responses to a pair of items may be locally dependent (responses to the items may be related to one another for a reason other than knee health).^22^

We then checked for differential item functioning (DIF) by age (< 60 years or ≥ 60 years) and sex using the *lordif R* package (version 0.3 to 3).^23^ This involved fitting logistic regression models that predicted response to an item, based on the combined score of all items. If the addition of either age or sex to these models improved the model fit by a Nagelkerke pseudo-R^2^ value of > 2%, we took this to suggest that age or sex significantly affected the relationship between knee health and item response. We did not treat the thresholds described in this section as hard binary cut-offs for performing IRT modelling, but rather as contextual evidence with which to make informed metrological judgements.

### Item response theory modelling

**Fig. 1:**
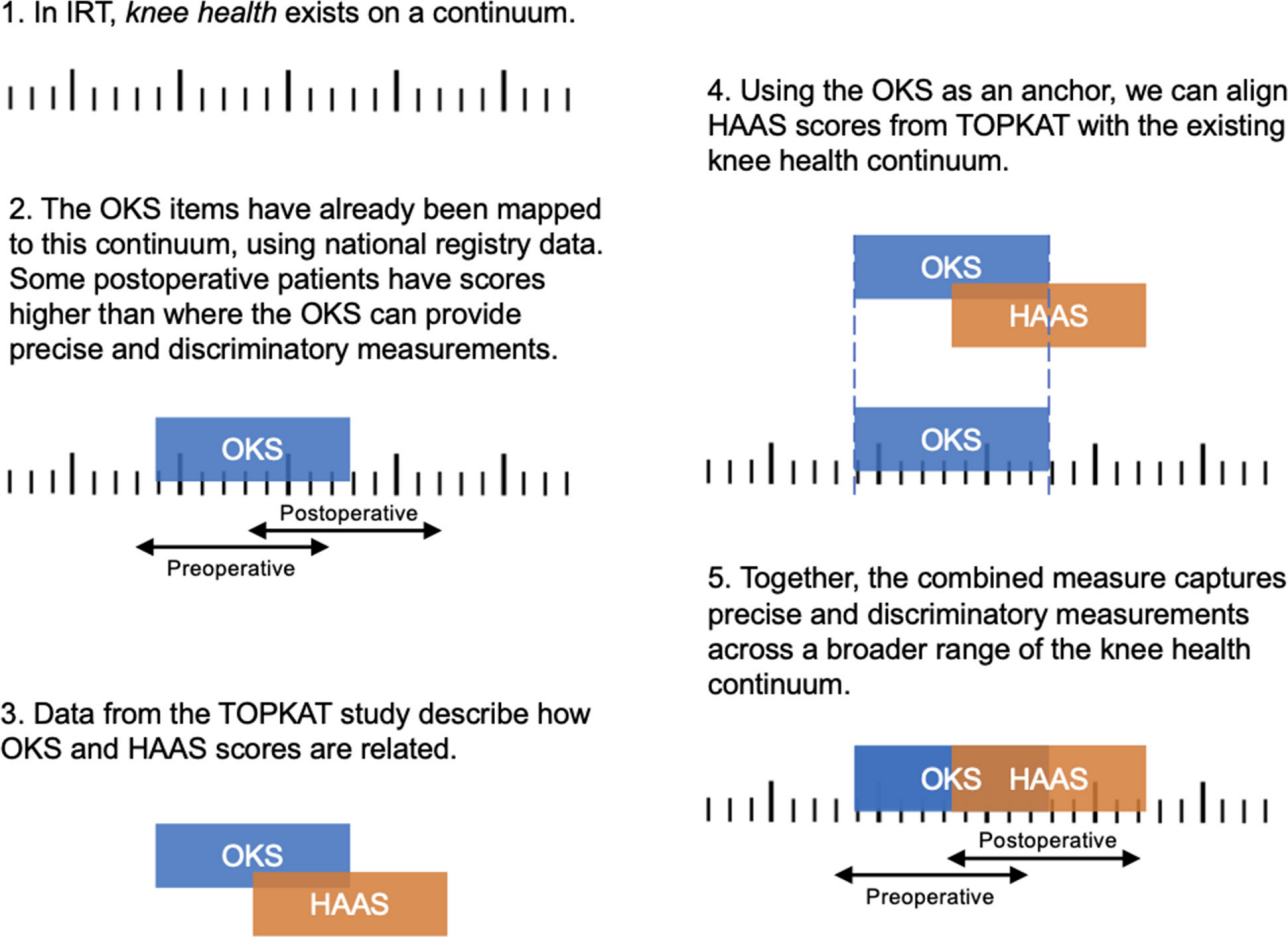
Schematic representation of study methods. HAAS, High Activity Arthroplasty Score; IRT, item response theory; OKS, Oxford Knee Score.

Following assumption testing, we fitted an item response theory model (specifically, a graded response model) to the combined OKS and HAAS items in the TOPKAT dataset. To do this, we used the Metropolis-Hastings Robbins-Monro algorithm in the *mirt R* package (version 1.36.1).^24^ When doing this, we constrained the parameters of the OKS items to exactly match the parameters that have previously been described (Figure 1). The HAAS item parameters were freely estimated. We plotted test-level information (which is closely related to measurement precision) across latent construct levels to understand how combining the PROMs might affect measurement precision in patients with high levels of knee health. Finally, we used our IRT model to calculate EAP sum-scores for the OKS and the HAAS,^25^ and presented these in a conversion table, allowing readers to map scores from each PROM onto a common scale.

For each individual at each timepoint, we used the conversion table to obtain common scale scores first using their OKS score, and then using their HAAS score. We compared the similarity of OKS- and HAAS-derived common scale measurements using distribution plots and Bland-Altman analysis.

## Results

### Participants

**Fig. 2:**
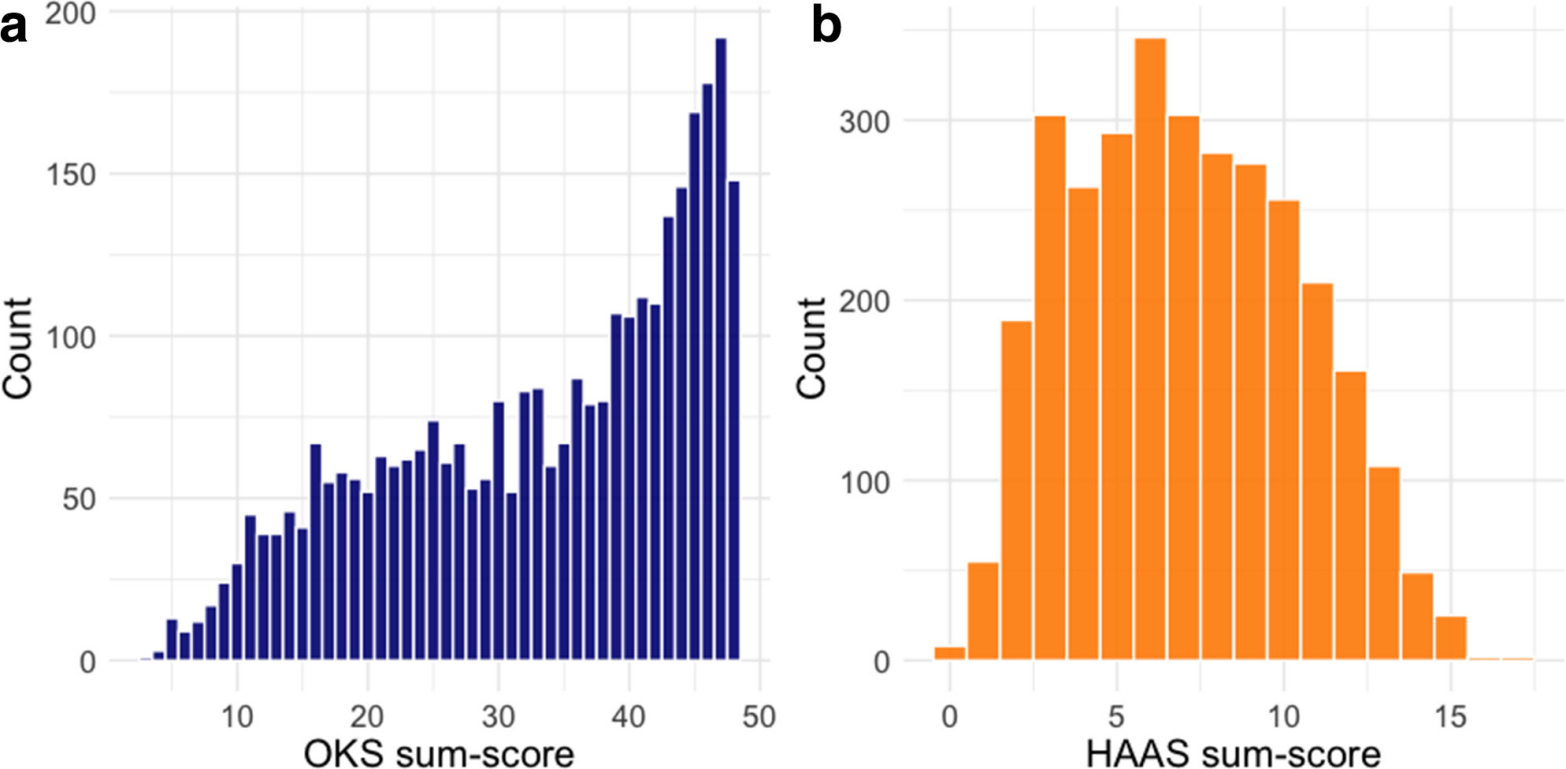
Histograms demonstrating the sum-score distribution of a) the Oxford Knee Score (OKS) and b) High Activity Arthroplasty Score (HAAS). The distribution of OKS sum-scores is positively skewed, while the distribution of HAAS has a slight negative skew.

We included 3,329 paired OKS and HAAS response sets from 528 participants. The mean age of these participants was 65 years (standard deviation (SD) 8.6); 306 respondents were male and 222 were female. The distribution of OKS and HAAS sum-scores (with repeated measures treated independently) are presented in Figure 2. The distribution of missing item responses is provided in Table I. In the 477 TOPKAT participants followed up at the five-year timepoint, 8.2% achieved the ceiling sum-score (48) of the OKS.

**Table I.** Distribution of missing item responses.

Item	Missing responses, n (%)
**HAAS items**	
Activity level	194 (6)
Running	165 (5)
Walking	144 (4)
Stair climbing	143 (4)
**OKS items**	
Kneeling	79 (2)
Walking	68 (2)
Shopping	66 (2)
Stairs	66 (2)
Transport	65 (2)
Work	65 (2)
Confidence	65 (2)
Night pain	63 (2)
Pain	58 (2)
Standing	57 (2)
Limping	57 (2)
Washing	53 (2)

HAAS, High Activity Arthroplasty Score; OKS, Oxford Knee Score.

### Assumption testing

The disattenuated correlation of OKS and HAAS sum-scores was 0.85. When combined, the item responses from the OKS and HAAS demonstrated good fit to the one-factor CFA model and all items had a Loevinger’s H_i_ ≥ 0.3. This suggests that the two PROMs measure a sufficiently similar construct for combination. Fit statistics and thresholds for the CFA are presented in Table II, and standardized pattern coefficients and Loevinger’s H_i_ statistics for each item are presented in Table III.

**Table II.** Fit of combined items to one-factor model.

Variable	RMSEA	SRMR	CFI	TLI
Threshold	< 0.060	≤ 0.080	≥ 0.950	≥ 0.950
Combined items	0.084	0.049	0.997	0.996

CFI, comparative fit index; RMSEA, root mean squared error of approximation; SRMR, standardized root mean square residual; TLI, Tucker-Lewis index.

**Table III.** Standardized pattern coefficients for the one-factor confirmatory model, Loevinger’s H_i_ values, and outfit, infit, and root mean squared error of approximation from the graded response model.

Item	Standardized pattern coefficient	Loevinger’s H_i_	Outfit	Infit	RMSEA
**OKS items**					
Walking	0.567	0.416	1.795	1.727	0.096
Standing	0.669	0.308	0.725	0.787	0.009
Limping	0.551	0.374	1.509	1.203	0.057
Kneeling	0.683	0.423	1.994	1.214	0.073
Transport	0.686	0.351	1.701	1.11	0.022
Work	0.746	0.417	0.899	0.912	0.018
Stairs	0.752	0.337	0.983	1.095	0.059
Give way	0.454	0.425	1.749	1.808	0.077
Shopping	0.798	0.469	0.999	0.959	0.026
Night pain	0.505	0.298	0.785	0.974	0.025
Pain	0.654	0.48	1.121	1.171	0.038
Washing	0.623	0.461	1.027	1.015	0.029
**HAAS items**					
Activity level	0.644	0.366	0.945	0.951	0.012
Running	0.588	0.333	0.891	0.962	0.006
Walking	0.637	0.397	0.935	0.938	0.011
Stair climbing	0.632	0.392	0.982	0.988	0.01

HAAS, High Activity Arthroplasty Score; OKS, Oxford Knee Score; RMSEA, root mean squared error of approximation.

The HAAS activity level and HAAS walking items shared a Yen’s Q3 residual covariance of 0.31. Yen’s Q3 was 0.22 between the OKS pain and night pain items, and 0.28 between the OKS limping and pain items. This suggests mild local dependence between responses to these items.

The kneeling item of the OKS showed uniform DIF by sex (Nagelkerke pseudo-R^2^ of 5%). In other words, for any given level of knee health, females find it more difficult to kneel and stand up again. This became negligible at the test-level, and is consistent with findings from the NHS England PROMs registry.^12^ We did not find DIF by sex or age in any other item.

### Model parameters

Table IV presents the graded response model parameters for the combined OKS-HAAS model. Parameters for the OKS items have been constrained to exactly match those derived from the NHS PROMs registry,^12^ and HAAS item parameters have been anchored to these. Item-level fit statistics for the model are presented in Table III.

**Table IV.** Graded response model parameters for the combined Oxford Knee Score and High Activity Arthroplasty Score scale. Values are presented as point estimates and 95% credible intervals.

Item	a	b_1_	b_2_	b_3_	b_4_	b_5_	b_6_
**OKS items**							
Walking	1.330 (1.321 to 1.339)	-1.920 (-1.932 to -1.907)	-1.103 (-1.111 to -1.094)	0.675 (0.667 to 0.682)	2.323 (2.308 to 2.339)		
Standing	1.951 (1.939 to 1.963)	-2.515 (-2.530 to -2.501)	-0.118 (-0.123 to -0.112)	1.299 (1.291 to 1.306)	2.858 (2.840 to 2.875)		
Limping	1.280 (1.271 to 1.290)	-0.299 (-0.306 to -0.292)	1.256 (1.246 to 1.266)	2.121 (2.106 to 2.136)	4.010 (3.976 to 4.043)		
Kneeling	1.387 (1.377 to 1.397)	-0.304 (-0.311 to -0.298)	1.195 (1.186 to 1.205)	2.949 (2.928 to 2.969)	4.468 (4.427 to 4.510)		
Transport	1.887 (1.875 to 1.899)	-3.756 (-3.787 to -3.725)	-0.984 (-0.991 to -0.977)	0.947 (0.940 to 0.954)	2.050 (2.039 to 2.062)		
Work	2.548 (2.532 to 2.563)	-1.406 (-1.413 to -1.399)	0.154 (0.149 to 0.159)	1.563 (1.556 to 1.571)	2.712 (2.696 to 2.728)		
Stairs	2.108 (2.096 to 2.121)	-2.119 (-2.130 to -2.108)	-0.382 (-0.388 to -0.377)	1.122 (1.115 to 1.129)	2.383 (2.370 to 2.396)		
Give way	1.501 (1.491 to 1.510)	-1.713 (-1.723 to -1.702)	-0.270 (-0.276 to -0.264)	0.651 (0.644 to 0.657)	2.171 (2.158 to 2.184)		
Shopping	2.222 (2.209 to 2.235)	-1.206 (-1.213 to -1.199)	-0.443 (-0.449 to -0.438)	0.690 (0.684 to 0.695)	1.673 (1.664 to 1.681)		
Night pain	1.221 (1.212 to 1.230)	-0.811 (-0.819 to -0.802)	0.484 (0.476 to 0.491)	2.003 (1.989 to 2.017)	2.624 (2.605 to 2.642)		
Pain	1.677 (1.665 to 1.689)	0.019 (0.013 to 0.025)	2.314 (2.300 to 2.329)	3.415 (3.389 to 3.441)	4.514 (4.464 to 4.564)		
Washing	1.475 (1.465 to 1.485)	-4.283 (-4.321 to -4.245)	-2.049 (-2.062 to -2.036)	-0.327 (-0.334 to -0.321)	0.734 (0.726 to 0.741)		
**HAAS items**							
Activity level	0.828 (0.780 to 0.876)	-3.420 (-3.761 to -3.079)	0.504 (0.383 to 0.625)	2.121 (2.021 to 2.222)	5.983 (5.770 to 6.197)	9.272 (8.712 to 9.832)	10.220 (9.446 to 10.993)
Running	1.022 (0.961 to 1.084)	2.064 (1.977 to 2.152)	4.453 (4.324 to 4.581)	7.680 (7.332 to 8.029)	9.535 (8.816 to 10.255)		
Walking	1.067 (1.015 to 1.120)	-1.215 (-1.364 to -1.067)	0.199 (0.096 to 0.301)	2.378 (2.295 to 2.461)	3.205 (3.115 to 3.294)	4.729 (4.600 to 4.859)	
Stair climbing	0.968 (0.906 to 1.030)	-3.366 (-3.709 to -3.023)	2.803 (2.710 to 2.897)	5.994 (5.786 to 6.202)			

"a" represents the discrimination parameter, "b1" difficulty parameter 1, "b2" difficulty parameter 2, and so on.

HAAS, High Activity Arthroplasty Score; OKS, Oxford Knee Score.

In Table V, we have provided EAP sum-scores and standard errors of measurement, corresponding to each possible sum-score in the OKS and HAAS. This can be used as a conversion table to translate scores from each instrument onto the common scale. To illustrate, a sum-score of 23 on the OKS is similar to a sum-score of 4 on the HAAS (EAP sum-scores of 0.48 and 0.47, respectively). Sum-scores of 11 or more on the HAAS represent levels of knee health higher than can be precisely measured by the OKS. The standard error of measurement gives an indication of the reliability of the measurement, with values < 0.55 considered desirable for group-level measurements.^26^

**Table V.** Sum-scores and corresponding expected a posteriori sum-scores of the Oxford Knee Score and High Activity Arthroplasty Score*,* based on the common scale.

OKS			HAAS		
Sum-score	EAP sum-score	SE	Sum-score	EAP sum-score	SE
0	-3.40	0.53	0	-3.13	1.17
1	-3.01	0.49	1	-1.95	1.16
2	-2.68	0.45	2	-0.96	1.10
3	-2.39	0.42	3	-0.18	1.00
4	-2.14	0.40	4	0.47	1.00
5	-1.93	0.39	5	1.08	0.99
6	-1.74	0.38	6	1.60	0.97
7	-1.56	0.37	7	2.15	0.95
8	-1.40	0.36	8	2.65	0.94
9	-1.24	0.36	9	3.12	0.93
10	-1.10	0.35	10	3.60	0.91
11	-0.96	0.35	11	4.11	0.87
12	-0.82	0.34	12	4.62	0.81
13	-0.69	0.34	13	5.03	0.72
14	-0.57	0.34	14	5.28	0.63
15	-0.44	0.34	15	5.36	0.60
16	-0.32	0.33	16	5.49	0.51
17	-0.20	0.33	17	5.57	0.46
18	-0.09	0.33	18	5.63	0.42
19	0.03	0.33			
20	0.14	0.33			
21	0.26	0.33			
22	0.37	0.33			
23	0.48	0.33			
24	0.59	0.33			
25	0.70	0.33			
26	0.81	0.33			
27	0.92	0.33			
28	1.03	0.33			
29	1.15	0.33			
30	1.26	0.33			
31	1.37	0.33			
32	1.48	0.33			
33	1.60	0.33			
34	1.72	0.33			
35	1.83	0.34			
36	1.95	0.34			
37	2.08	0.34			
38	2.20	0.34			
39	2.33	0.35			
40	2.47	0.35			
41	2.61	0.36			
42	2.76	0.37			
43	2.92	0.38			
44	3.08	0.40			
45	3.26	0.31			
46	3.46	0.43			
47	3.67	0.46			
48	3.9	0.50			

EAP, expected a posteriori; HAAS, High Activity Arthroplasty Score; OKS, Oxford Knee Score; SE, standard error of measurement.

**Fig. 3:**
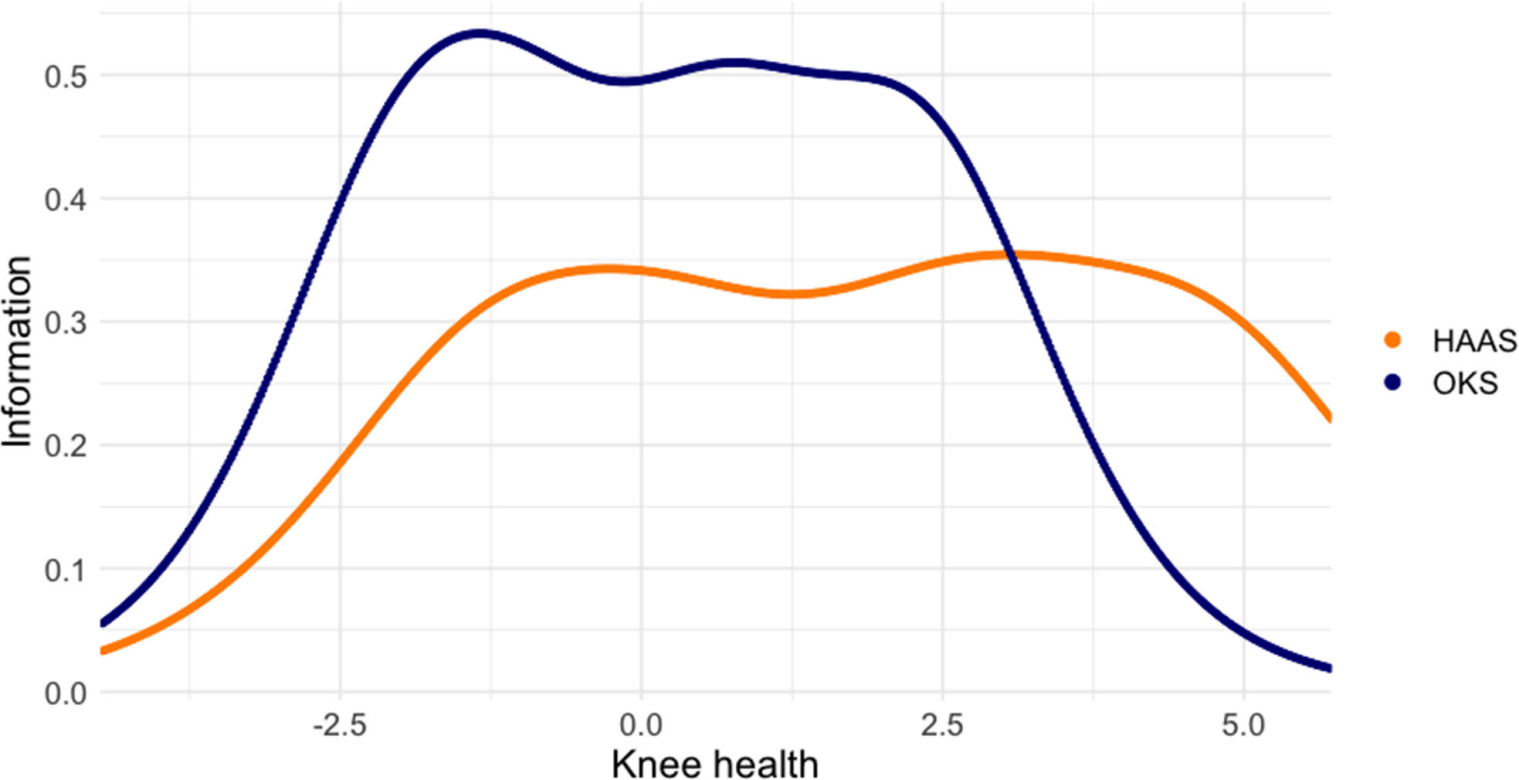
Information provided by the Oxford Knee Score (OKS) stair climbing item and the High Activity Arthroplasty Score (HAAS) stair climbing item across knee health levels. At knee health levels over three logits, the HAAS item provides more precise measurement than the OKS item.

The ability for HAAS items to extend the discriminatory range of the OKS is illustrated for the stair climbing items in Figure 3. The most positive response to the OKS stair climbing item indicates that a respondent can easily walk down a flight of stairs, while the most positive response to the HAAS stair climbing item indicates that a respondent can climb stairs two at a time. In this figure, information relates to the precision of measurement that can be achieved by each item. The OKS item provides higher measurement precision than the HAAS item (more discriminatory measurement) with respondents who have a knee health level under three logits. However, at higher levels of knee health, the HAAS item becomes more discriminatory than the OKS item.

**Fig. 4:**
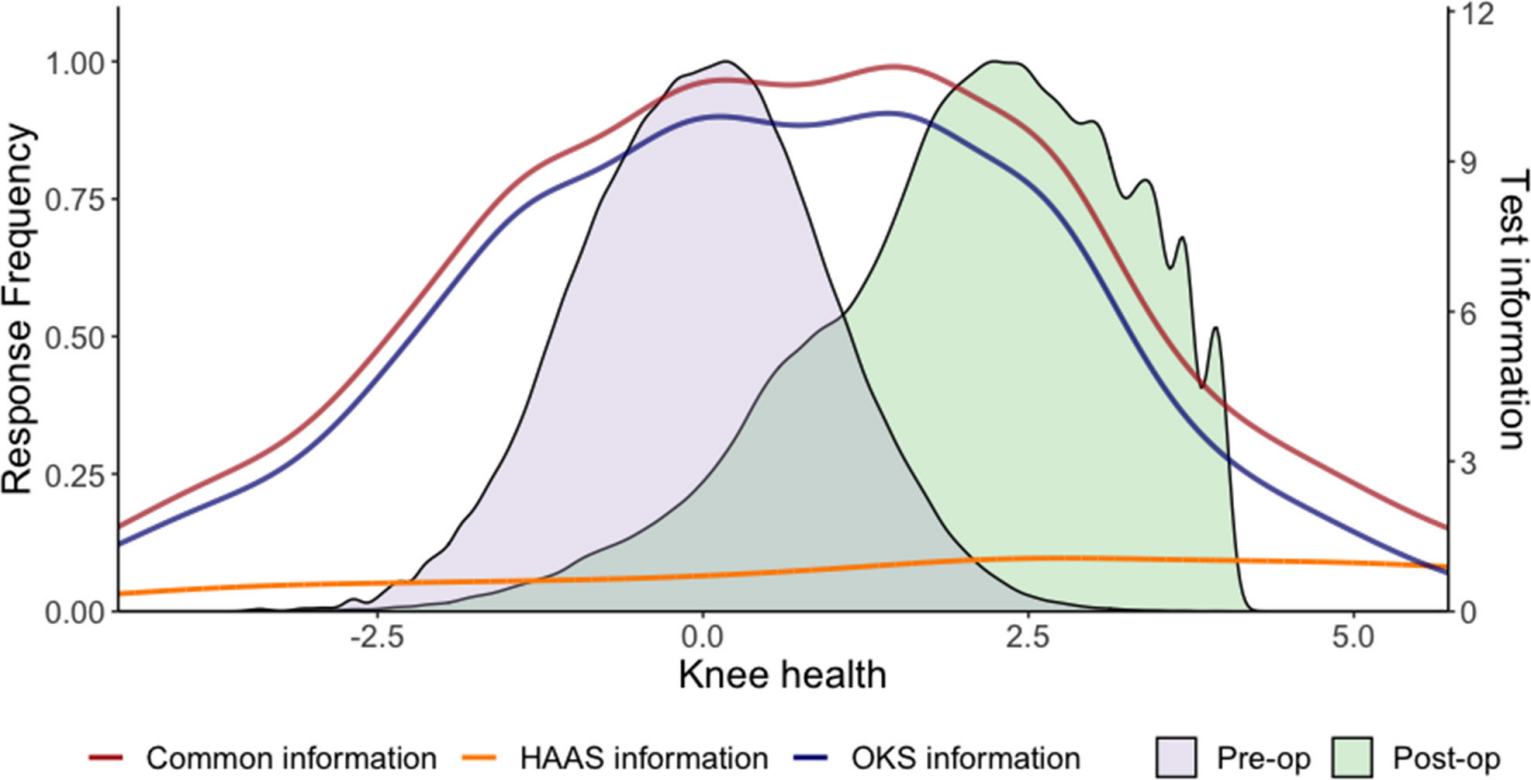
Information provided by the Oxford Knee Score (OKS), the High Activity Arthroplasty Score (HAAS), and the combined measure. The orange line represents the test-level information of the HAAS across different levels of knee health, the blue line represents the test-level information of the OKS, and the red line represents the test-level information of both instruments combined. Information levels greater than 9.8 are considered to indicate excellent measurement precision. For reference, the score distributions of preoperative and postoperative arthroplasty patients in the NHS patient-reported outcome measures registry have been included and shaded magenta and green, respectively. The combined instrument provides more precise measurement than the OKS alone, within a knee health range that is relevant to patients undergoing arthroplasty.

Together, the four HAAS items provide relatively low-precision measurement, compared to the 12 OKS items (Figure 4). However, when the HAAS items are used together with the OKS items, the combined instruments provide higher-precision measurement than the OKS items alone, across all levels of knee health, and particularly at higher levels, where many postoperative arthroplasty patients are located. This suggests that when both instruments are used together, and scored with the parameters presented in Table IV, more precise and discriminatory measurement can be achieved in knee arthroplasty than by using the OKS alone. For context, information levels higher than 9.8 are considered to indicate excellent measurement precision at the individual level.^26^

**Fig. 5:**
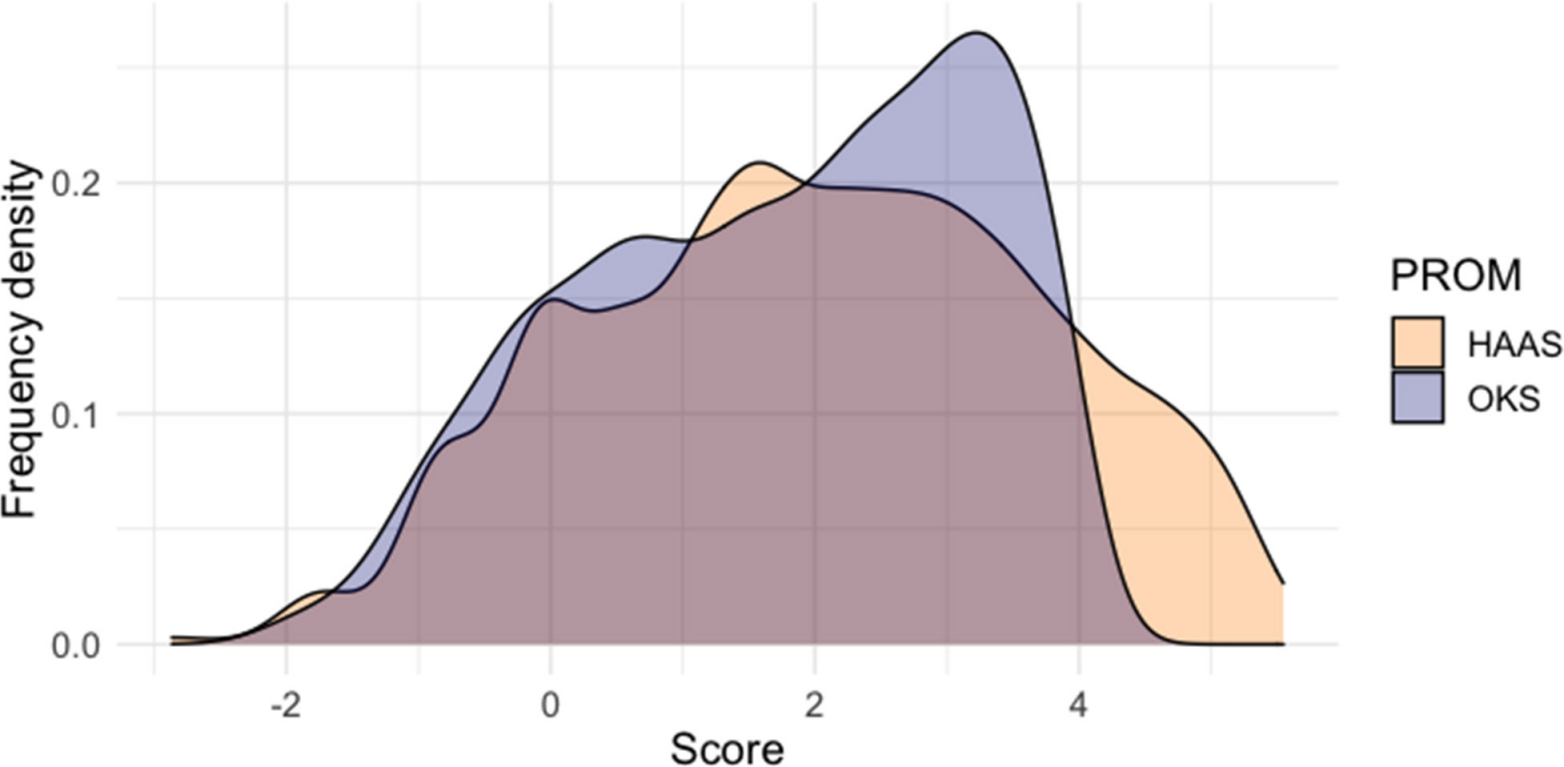
Distribution of paired common scale scores derived from the Oxford Knee Score (OKS) and High Activity Arthroplasty Score (HAAS). PROM, patient-reported outcome measure.

**Fig. 6:**
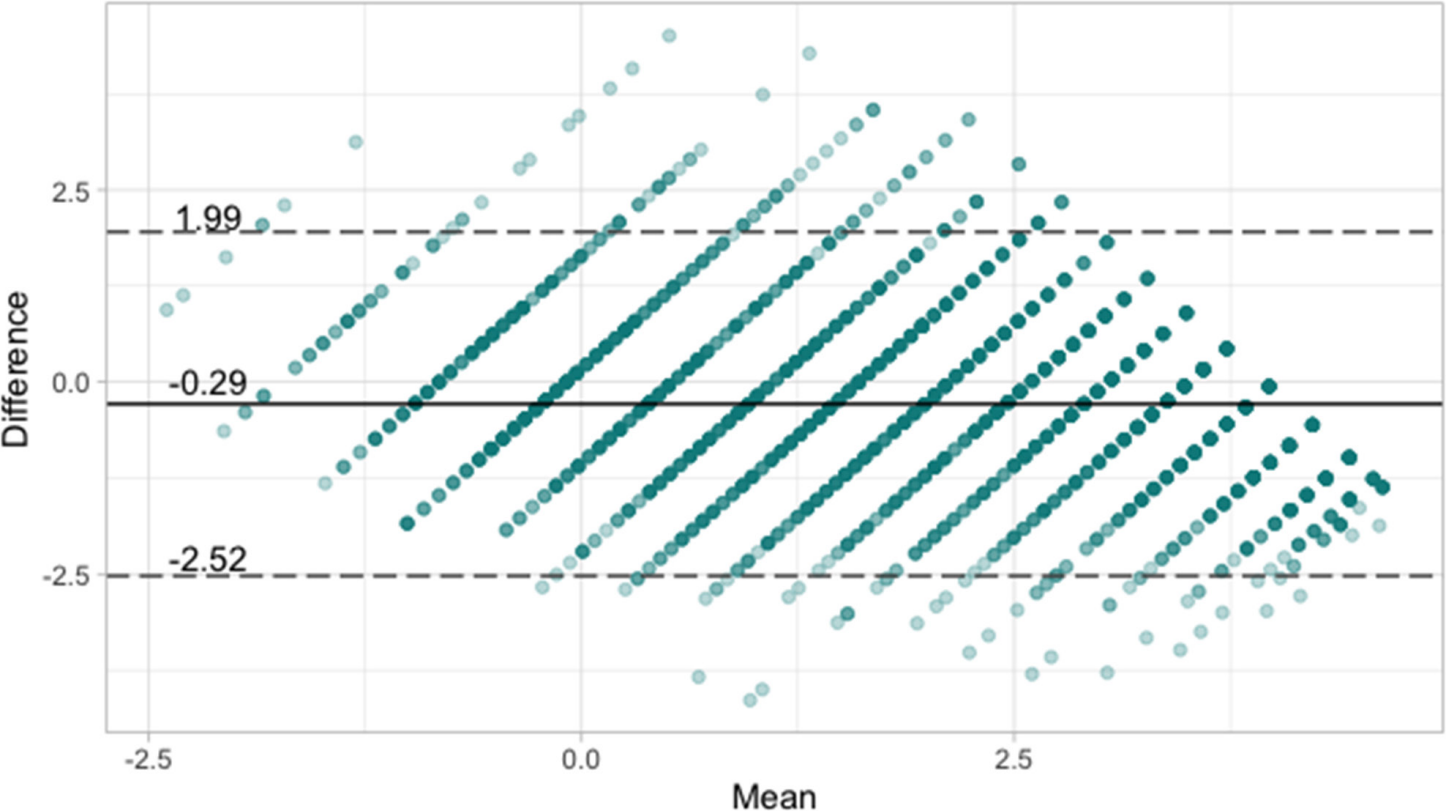
Bland-Altman plot illustrating the agreement of Oxford Knee Score (OKS)- and High Activity Arthroplasty Score (HAAS)-derived common scale measurements. The solid horizontal line represents the mean difference between OKS- and HAAS-derived common scale measurements (mean of HAAS-derived score subtracted from OKS-derived scores, -0.29). The horizontal dashed lines represent the 95% limits of agreement (95% of paired common scale scores fall within these limits).

The distribution of OKS- and HAAS-derived common scale measurements was similar (Figure 5). Bland-Altman analysis (Figure 6) showed significant disagreement between OKS- and HAAS-derived common scale measurements at the individual respondent level (95% limits of agreement ranging from -2.52 to +1.99 logits), but high agreement at the group level (mean error of -0.29 logits).

## Discussion

This exploratory study has demonstrated that it is possible to map the OKS and HAAS onto a common knee health scale, using IRT. Our conversion table allows readers to map individual scores from either PROM onto the common scale, and may assist in the pooling of patient-level data from studies that report the scores of either PROM.

The item parameters presented in this paper could be used to derive more precise measurements from the two instruments combined than those derived from the OKS alone, particularly in the postoperative setting. For now, this can be achieved by administering both PROMs and then using IRT scoring software (such as the *mirt* package in R, which is free to use) to score the instruments with the parameters provided in Table IV. A more appealing solution, which should be explored in future, may be to develop a computerized adaptive test (CAT) that can pick the most appropriate items to administer to an individual, from either PROM, based on the answers provided to previous items in the assessment. By selecting only the most relevant items for an individual, CAT may be able to achieve high levels of precision with fewer items than the two PROMs combined. Simulation studies could test this hypothesis, using freely available CAT simulation software, such as the *mirtCAT* R package,^27^ and the item parameters provided in this paper. This would complement ongoing efforts to shorten and personalize the Oxford scores through CAT, which have shown very promising results so far.^28,29^

The most similar work to this has been the mapping of KOOS and PROMIS physical function scores onto a common scale, which was conducted as part of the PROsetta stone initiative.^7^ Similarly to our findings, the authors showed that the KOOS and PROMIS physical function scales were sufficiently unidimensional for combination (they both measure the same, or a very similar, knee health construct). The item content of the OKS, HAAS, and the KOOS and PROMIS physical function scales is similar in terms of face validity. Given that the OKS and HAAS can be considered unidimensional, and the KOOS and PROMIS physical function scales can be considered unidimensional, it is likely that all four instruments reflect the same (or a very similar) knee health construct, and could be combined onto a common scale. This would require paired responses from either the OKS or HAAS, and either the KOOS or PROMIS physical function scales.

While this study provides a promising proof of concept, it has notable limitations. First, we were not able to externally validate our findings with the data resources available, and for that reason we would term this work explorative. The external validity of our conversion table (Table IV) should be tested with independent, patient-level data. To do this, a validation study might aim to predict the scores of one PROM from the other, and quantify prediction error at the individual level. The OKS ceiling effect complicates the use of group averages for this purpose, and while some existing studies have published paired OKS and HAAS scores averaged across the group level, we found no prior studies reporting patient-level sum-scores for both instruments. The agreement of OKS- and HAAS-derived common scale scores presented in this paper (see Figure 5 and Figure 6) was estimated from the same dataset used to derive HAAS item calibrations, and for that reason it cannot be considered a true form of validation.

Second, we chose to treat within-person repeated measures independently to ensure that a broad range of response options were included (preoperatively, patients typically achieve only low HAAS scores). This may have inflated fit statistics, reduced the size of the confidence intervals surrounding item parameters, and introduced bias (for example, if 528 of the respondents in this study interpret and respond to the PROMs each time in a way that is in keeping with the IRT model, but differs from the broader population). It also assumes that respondents interpret and respond to the items in the same way at each timepoint (i.e. a lack of response shift). Readers should keep this in mind when interpreting our findings. In the specific case of the OKS and HAAS, this limitation may be unavoidable, as patients awaiting knee arthroplasty are unlikely to achieve the highest scores in HAAS items, and response data are needed for item parameterization.

Third, the HAAS itself demonstrated relatively low-precision measurement in our sample. For context, an information level of > 5.0 approximately equates to a marginal reliability of 0.80, which is considered by some to be the minimum level that is acceptable for group-level measurement.^26^ In this study, we found that the HAAS had a considerably lower precision than this across the entire range of knee health measurements (Figure 4). This might not be surprising, as the HAAS was not developed to provide high-precision measurement in a general arthroplasty population. Cronbach’s alpha for the HAAS was 0.80, which is lower than previous estimates reported in the literature,^11,30^ but this should be interpreted cautiously, given our decision to treat observations independently. Sum-scores and EAP sum-scores for the HAAS, including those derived from our conversion table, should be interpreted with this in mind. While linked PROM scores can be used to compare groups of participants, they are not necessarily appropriate for comparing individuals,^31^ and our Bland-Altman analysis (Figure 6) suggests that individual-level common scale scores may contain large errors.

The combined items performed well, but not perfectly, against the IRT assumption tests reported in this paper. We found mild local dependency between the HAAS activity level and walking items, between the OKS pain and night pain items, and between the OKS pain and limping items. However, this was not severe, and our residual covariance statistics may also have been influenced by our decision to treat within-person measurements independently. We found DIF by sex in the OKS kneeling item, which is consistent with the original IRT modelling study for the instrument, performed on a dataset of over 350,000 response sets. The effect of this was negligible at the test level, when all OKS items were combined.^12^ The relatively low discrimination parameters of the HAAS items, and the disattenuated sum-score correlation of 0.85, might suggest a degree of multidimensionality between the PROMs. An emerging technique for PROM linkage called ‘calibrated projection’ has demonstrated accuracy gains over unidimensional fixed parameter calibration in simulation studies where a degree of multidimensionality exists between measures.^32^ The potential to improve this mapping with calibrated projection could be explored once external validation data become available.

Today, clinicians and researchers are faced with a challenging decision when selecting a PROM to measure the impact of knee arthritis and its treatment. In theory, many of these instruments could be mapped onto a common IRT scale which would support the standardization of outcome reporting and pooling of results, minimize research waste, and deliver potential gains in measurement range and precision. To achieve this goal, we should make paired response data freely available, continue to perform mapping studies such as this, and validate the results with independent, patient-level data.
